# Contemporary Evidence of CAD-CAM in Dentistry: A Systematic Review

**DOI:** 10.7759/cureus.31687

**Published:** 2022-11-20

**Authors:** Mahesh Suganna, Hina Kausher, Sara Tarek Ahmed, Haifa Sultan Alharbi, Bushra Faraj Alsubaie, Aruna DS, Shaista Haleem, Abbasi Begum Meer Rownaq Ali

**Affiliations:** 1 Prosthodontics, College of Applied Medical Sciences, Riyadh Elm University, Riyadh, SAU; 2 Prosthodontics/Dental Lab Technology, College of Applied Medical Sciences, Riyadh Elm University, Riyadh, SAU; 3 Prosthodontics, College of Dentistry, Riyadh Elm University, Riyadh, SAU; 4 Dental Lab Technology, College of Applied Medical Sciences, Riyadh Elm University, Riyadh, SAU; 5 Dentistry/Dental Public Health and Tobacco Cessation, Private Consultancy, Bengaluru, IND; 6 Aesthetic and Restorative Dentistry, College of Applied Medical Sciences, Riyadh Elm University, Riyadh, SAU

**Keywords:** computer-aided manufacturing, computer-aided design, analysis, systematic review, future of dentistry, dentistry, cad/cam

## Abstract

In this systematic review, we compare the quality and accuracy of computer-aided design (CAD)/computer-aided manufacturing (CAM) techniques currently employed in dentistry with those of traditional materials. Published literature on the study topic was searched in the online MEDLINE, PsycINFO, PubMed, and Cochrane library databases and the database of Indian Council of Medical Research. For this systematic review, the Preferred Reporting Items for Systematic Reviews and Meta-Analyses (PRISMA) framework was applied for the assessment of studies fit for investigation. Of the total 103 papers searched, 54 underwent in-depth evaluation. Using criteria for what to include and what to leave out, we chose research that was relevant to our review and narrowed it down to 14 papers that met the review's guidelines. According to our findings and the analysis of the chosen articles, the prospects and current advances of CAD/CAM technology are fascinating and are revolutionizing the field of dentistry. Even though researchers are excited, it is important to make sure that different materials have been tested and looked at well enough before making firm claims and choices to replace materials that have been made in the past. Based on previous research, it has been determined that the CAD/CAM methodology used in the dental field is the most popular method at the moment since it is quick, simple, and efficient. Since there are different kinds of CAD/CAM systems, it is very important to choose the right one and come up with a good plan for treating a patient.

## Introduction and background

Over the past 20 years, computer-aided design (CAD)/computer-aided manufacturing (CAM) has become more common in dentistry [1]. Inlays, onlays, veneers, crowns, fixed partial dentures, implant abutments, and even full-mouth reconstruction can be done with this method. It is used in dental offices and dental laboratories [2]. CAD/CAM is also used in orthodontics. The development of CAD/CAM technology was motivated by three challenges. The first challenge was making sure the fix was robust enough, especially for posterior teeth. The creation of restorations that seemed natural presented the second challenge. The final challenge involved making tooth repair easier, quicker, and more accurate. Due to CAD/CAM technology, patients occasionally get restorations on the same day [3]. Dentists and laboratories may use the new technology in a variety of ways. Dentists can either perform their own internal CAD and milling, or they can build a digital image and submit it to a lab for manufacturing restorative materials. When laboratories get a digital impression, they can use the information to create a stone model, which can then either be used as is for traditional production or rescanned for milling [4]. As an alternative, the lab can complete all of the design work online using the photos obtained.

 The design and production of dental restorations, particularly dental prostheses like crowns, crown lays, veneers, inlays and onlays, fixed bridges, dental implant restorations, removable or fixed dentures, and orthodontic appliances, are improved by the use of CAD/CAM in the field of dentistry [5]. CAD and production were developed in the 1960s for use in the automobile and aerospace industries. It is conceivable to pinpoint the 1980s as the decade when CAD/CAM technology first entered the dental sector [6]. CAD/CAM technology was introduced to dentistry in 1989 by Mormann & Brandestinni in Germany, and it was developed for the in-office fabrication of ceramic restorations specifically to enable the dentist to complete one or multiple ceramic restorations chairside, in a single appointment [7]. These CAD/CAM technologies can be used to machine as well as precisely and accurately create various restorations and dental prostheses. Over the past 25 years, there has been a substantial increase in the utilization of CAD/CAM technology [8]. The development of CAD/CAM technology in dentistry has significantly altered treatment plans and the production of prosthetics. In this systematic review, the history and basic operation of CAD/CAM in dentistry were covered, and we sought to confirm our initial hypotheses of whether CAD/CAM usage would enhance the quality of dental instruments/devices in comparison to traditional methods that exist.

## Review

This systematic review was performed using the Preferred Reporting Items for Systematic Review and Meta-Analysis (PRISMA) strategy and in accordance with rules from the Cochrane group. Approval was given by the Institutional Ethics Committee Review Board (IRB) of Riyadh Elm University, Riyadh, Saudi Arabia (approval number: FRP/2022/461/826/787).

**Figure 1:**
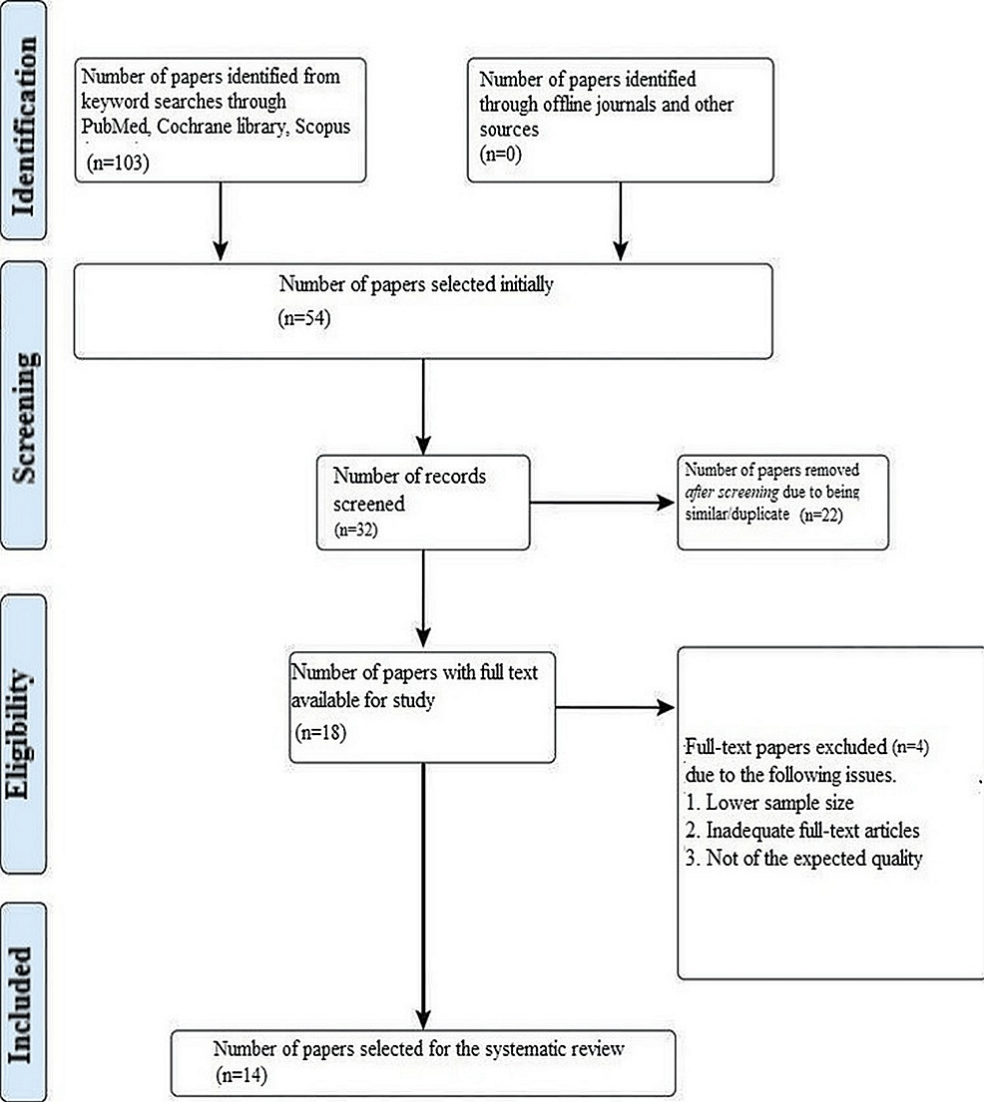
Representation of the selection of articles through the PRISMA framework PRISMA: Preferred Reporting Items for Systematic Reviews and Meta-Analyses

As per our initial hypothesis, this systematic review set out to examine the current state of CAD/CAM in dentistry and how technology has affected and will continue to influence this industry. After a thorough search of the online journals, 103 documents were found in total, and 54 of the papers were initially chosen. Twenty-two similar or duplicate publications were then removed, leaving 32 distinct papers that were initially available. Following a review of the submissions' abstracts and titles, an additional 18 articles were disqualified. In the end, 14 papers were selected, which satisfied the inclusion and exclusion criteria, including study articles and randomized and non-randomized control trials (Figure 1).

In terms of the inclusion criterion employed for our investigation, we considered articles that had full-text availability and were written in English, in vitro studies, comparative studies, systematic reviews with complete meta-analysis of their investigations, and case-control trials. Also, since the literature available on this topic is quite scant in volume, we did not limit our search in terms of the time period when the studies were published, i.e., we took into account all the papers that were published with context to our topic (where the number of papers itself was found to be quite sparse in number).

The following were eliminated from the scope of our systematic review based on the exclusion criterion: incomplete data, seminar presentations, academic articles, placebo-controlled studies, opinion articles, and scoping reviews. Studies that used a placebo were excluded from the analysis. Reviews of the literature and cases that were published in languages other than English were also omitted. The following databases were scoured for studies about the implementation of CAD/CAM in dentistry: MEDLINE, PsycINFO, PubMed, the database of the Indian Council of Medical Research, and Cochrane. Other published studies were found by scanning the reference lists of pertinent papers.

Table 1 includes the findings of the systematic review as well as information on the 14 studies that were picked for the review.

**Table 1:** Tabular representation of the studies used for this systematic review CAD/CAM: computer-aided design/computer-aided manufacturing; CRM: CAD reference model; 3D: three-dimensional; ZrO: zirconium dioxide; FDPs: fixed dental prostheses; MIL: milling; SLS: selective laser sintering; SLM: selective laser melting; CAS: conventional melting-casting technology; LV: linear polarization; CV: cyclic voltammetry; EIS: electrochemical impedance spectroscopy; CA: chronoamperometry; CP: chronopotentiometry; STL: standard tessellation language

Author and year of study	Total sample size	Study design	Study description	Study inference
Awada et al., 2015 [9]	25	In vitro study	Polished 4x1x13.5 mm bars (n=25) were produced from milling blocks of each tested material that were trimmed to the required dimensions. The bars were subjected to a three-point flexural test on a 10-mm span at a crosshead speed of 0.5 mm/min. 42 conventional monolithic crowns were also manufactured, 7 of each material. The quality of the margin edges was examined using macrophotography and optical microscopy, which provided both a qualitative visual assessment and a measurement of the degree of pre-existing roughness.	The recently developed polymer-based CAD/CAM materials demonstrated considerably higher flexural strength and resilience modulus values together with lower flexural modulus values when compared to the tested ceramic or hybrid materials.
de Araujo et al., 2015 [10]	4	Comparative study	CAD/CAM technology was used to create four samples made of zirconia and four samples made of cobalt-chrome. The control groups consisted of four cobalt-chrome one-piece cast samples with a total of 12 constructed frameworks.	When compared to traditionally manufactured frameworks, CAD/CAM-made frameworks demonstrated better passivity.
Homaei et al., 2016 [11]	24	In vitro study	When compared to traditionally manufactured frameworks, CAD/CAM-made frameworks demonstrated better passivity. The flexural strength of each CAD/CAM ceramic after three-point bending was calculated using Weibull analysis. The elastic modulus was determined using the load-displacement curve. These ceramics (n=24) were subjected to sinusoidal loading with a frequency of 8Hz and a minimum load of 3N utilizing three-point bending cycles in order to test for cyclic fatigue. The fatigue limitations of these ceramics were also predicted.	Zirconia was discovered to have mechanical properties that were noticeably superior to those of the two other studied ceramics, including the CAD one, and its fatigue limit was found to be almost half of the mean flexural strength for all tested ceramics.
Jeong et al., 2018 [12]	40	Comparative study	A facsimile of a real tooth was first scanned using an oral scanner. A CRM was made utilizing the scan data after it was obtained, and a total of 10 models were then produced using either the milling method or the 3D printing method. The 20 models were scanned with a desktop scanner before the CAD test model was produced.	Although the accuracy of the 3D printing process is higher than that of the milling approach, neither technique is yet a viable option for creating prosthetics.
Katsoulis et al., 2014 [13]	17	In vitro study	Using computer-aided design and manufacture, ZrO frameworks for a screw-retained 10-unit implant-supported reconstruction on six implants were made. The implant platform and the resin pattern for the cuspid-supporting framework were digitalized using a laser (ZrO-L, N = 6) and a mechanical scanner (ZrO-M, N = 5). The control was composed of laser-scanned titanium (TIT-L, N = 6) and cast alloy frameworks (Cast, N = 5) that were both built using the same model and resembled ZrO frameworks in appearance.	Using optical and tactile scanners along with CAD/CAM technologies, it is able to create extremely accurate long-span screw-retained ZrO implant reconstructions.
Nejatidanesh et al., 2016 [14]	40	Comparative in vitro study	An abutment and its analogue fixture were positioned in a maxillary cast's left central incisor region. Four distinct types of implant-supported single restorations were developed on the abutment (n = 10). The internal and marginal gaps of the research groups were assessed using the replica method and a stereomicroscope.	Despite the marginal and internal gaps of the tested implant-supported restorations falling within the range that is clinically acceptable, it was discovered that single crowns produced utilizing CAD/CAM technology offer a better marginal fit.
Padros et al., 2020 [15]	36	In vitro study	In order to replace a missing (pontic) tooth, a fixed partial denture with three pieces was constructed on natural abutments. This clinical case served as the inspiration for the master model. Nine scans were conducted by each test subject, and 36 copies were produced using dental CAD-CAM software. The same batch of chrome-cobalt (CrCo) alloy was used to make both the three-axis and five-axis frames. No cement used to secure the frames.	There are no statistically significant differences between the two CAD-CAM systems under examination for the flexural strengths of these restorations, which range from 6500 to 7000 N. The more axes, since the roughness is reduced, the better the surface quality and vertical fit, according to this contribution.
Presotto et al., 2017 [16]	20	In vitro study	Using CAD-CAM (n = 10) and overcasting (n = 10) procedures, three-unit FDPs supported by implants were constructed. The frames were waxed to resemble a mandibular first premolar to first molar FDP using clouded mini-abutment cylinders. The wax designs were either overcast (overcast experimental group) or scanned to construct the frameworks (CAD-CAM control group). All of the frameworks were constructed from cobalt-chromium (CoCr) alloy.	The marginal fit and stress values shown by the CAD-CAM and overcasting techniques are equivalent for three-unit FDP frameworks. The worse marginal fit of the frameworks results in increased stress on the implant-supported system.
Savencu et al., 2018 [17]	4	Comparative study	On samples made using CAD/CAM MIL, SLS, SLM, and CAS, various electrochemical techniques, such as LV, CV, and EIS, and chronoelectrochemical studies were used (CA and CP).	The outcomes of computer-aided processing technologies were promising and provided a workable substitute to traditional manufacturing processes for the metallic frames used in dental prostheses.
You et al., 2021 [18]	20	In vitro study	A maxillary complete edentulous cast was replicated with a silicone material, scanned with a lab scanner, and saved as an STL file in order to construct a master gypsum cast. This was exported into a computer-aided design application in order to develop and store the trial denture. Using the set layer thicknesses, 20 dentures were created.	The trueness of the intaglio and cameo surfaces for the 50-m and 100-m stereolithography experimental dentures differed noticeably. But whereas the intaglio surface did not show a statistically significant difference in precision, the cameo surface did.
Tasaka et al., 2021 [19]	-	In vitro study	3D scanning was done for edentulous jaw models. The wax denture underwent 3D scanning after the teeth were placed on it. Heat-cured dentures were produced using heat-cure polymer resin. AM dentures were created using UV-exposed acrylic resin and were based on data that were collected by taking wax denture data out and adding model data.	The maxillary dentures manufactured by additive manufacturing showed a greater displacement compared to heat curing.
Wemken et al., 2020 [20]	16	In vitro study	16 plaster models of edentulous maxilla were created using a silicone mold, and they were afterwards scanned for digitization on a desktop computer. After manufacturing, hydrothermal cycling, and three, four, and six cycles of 640 W, 6 min microwave sterilization, a total of 48 samples were scanned. The overall intaglio surface, the palate, the alveolar ridge, and the border seal region were measured using an inspection programme to determine the root mean square estimation, positive and negative mean deviations, and the 3D surface deviation.	Subtractive manufacturing, followed by injection molding and stereolithography printing, produces the most accurate denture foundations. Only denture bases made using stereolithography remained dimensionally stable after microwave sterilization.
Yoon et al., 2020 [21]	9	Clinical study	Nine patients with a total of 12 missing arches were included in this study (7 maxillary and 5 mandibular). The full denture bases with occlusion rims for each edentulous arch were built using three different techniques. The denture bases were constructed by subtracting digitally from a virtual denture foundation with an occlusion rim. The bases of the entire denture were inserted intraorally using an indication that had been applied on the intaglio surfaces.	The digital light processing denture base demonstrated clinically acceptable tissue surface adaptation to both edentulous the maxilla and mandible when compared to other methods of denture base fabrication, and the denture base was likely to exhibit intimate tissue adaptation in the stress-bearing areas of maxillary arches.
Yuce et al., 2019 [22]	12	Comparative study	Thirty heat-pressed and 31 CAD/CAM porcelain laminate veneers were produced for twelve patients. Each veneer was silicone-replicated. Replicas were split into four portions to measure the changes to the veneers. A stereomicroscope was used to measure three spots for marginal and nine spots for internal alterations on replicas at a 40x magnification. Prior to cementation as well as 6, 12, 18, and 24 months later, clinical evaluations were done.	The fabrication process, whether CAD/CAM or heat-pressed, had no impact on the porcelain laminate veneers' internal or external adaptation. The results showed that both fabrication techniques worked well after two years of clinical use.

Discussion

According to the articles we chose and our analysis, CAD/CAM technology seems to have the edge over traditional methods/materials, especially with respect to aesthetic fixed restorations. The application of this technology offers high quality, professionalism, and financial gain, as well as a continual rise in "new" and contented patients. Any advancement in information and communication technology also has applications in a number of medical specialties, including dentistry. This study provides more proof that IT applications in dentistry are essential. Rapid diagnosis, timely and suitable treatment, monitoring of therapeutic effects, and achieving a high degree of aesthetics in all areas of dentistry and medicine as needed will all be significantly impacted by future improvements in information and communication technology.

Today, almost every aspect of dentistry uses CAD/CAM technology. According to a study on maxillo-facial prostheses, hand-carved prosthetic ears produce different results compared to those created with CAD/CAM technology. The latter is superior to the hand-carved prosthetic [23]. Therefore, prosthetic ears and nasal prostheses are made using CAD/CAM technology [24]. Additionally, certain bony deformities brought on by trauma or tumor excision are being addressed by implant creation utilizing CAD/CAM. Due to their aesthetics, clear aligners have gained enormous popularity in orthodontics. Metal brackets and cables are no longer necessary to be seen on patients. Again, CAD/CAM deserves praise. Due to the fact that it is invisible, the lingual bracket procedure is highly common [19]. The wires and bracket systems for the lingual bracket system are produced using CAD/CAM fabrication [25]. Orthodontic micro-implants can be positioned with the use of CAD/CAM technology. According to some of our chosen research, zirconia is the material that is most usually used to create crowns and bridges using CAD/CAM. However, metal and porcelain crowns and bridges can also be made using CAD/CAM. The research claims that zirconia has respectable clinical outcomes. Restorations like inlays, onlays, and veneers are also produced using CAD/CAM, as was observed in a study by Williams et al. [26] who used patient-fitted chromium cobalt removable partial denture framework produced by computer-assisted design, computer-assisted manufacture and rapid prototype technologies, a methodology similar to the one used in the study that we selected for our review done by Presotto et al. [16], the only difference being that this study manufactured fixed partial denture using CAD/CAM technology.

The fabrication material is one factor that affects the particulars of CAD/CAM repairs. Both patients and dentists find the procedure described to be very appealing because of the advantages that all of the materials used to share. Batson et al., in their study, recruited 22 individuals in need of posterior complete coverage crowns after which they were randomized to one of three groups: metal ceramic, lithium disilicate, and monolithic zirconia [27]. It was found that the average horizontal marginal discrepancy was significantly different between lithium disilicate and zirconia crowns, with zirconia crowns having the least amount of horizontal marginal discrepancy, showing that CAD/CAM-generated restorations for posterior teeth made from different materials had acceptable clinical results. The use of zirconia in this study mirrors three investigations that we used in our review, all of them showing promising results with respect to CAD/CAM manufacturing [10,11,13]. The clinical study done by Yoon et al. [21] also had the involvement of nine patients in need of complete prostheses, again mirroring the investigation by Batson et al. [27] in terms of selecting individuals for demonstration of this technology. Randomized clinical investigations have found that digital scanning is more efficient and more comfortable than traditional impressions [28]. A review, similar to ours, aimed to review the state-of-the-art of CAD/CAM all-ceramic biomaterials [29], and the authors observed that developments in CAD/CAM technology have catalyzed research in all-ceramic biomaterials and their applications. This inference was shared by the studies by Awada et al. [9] and Homaei et al. [11] that we selected for our review, which showed the use of ceramic in the fabrication of prostheses using CAD/CAM technology. It is quite remarkable that all the inferences that we observed after reviewing the studies that we selected for our review [9-22] are in agreement with investigations done by Papadiochou et al. [30], Ahmed et al. [31], and Bilgin et al. [32]. An inference that these three studies shared is that by improving the quality of the instruments and systems utilized, CAD/CAM in several dental specialties has opened up new potential for providing high-quality dental care while also theoretically increasing patient convenience [30-32]. These reviews also emphasize the importance of accuracy which is a crucial component as well. Single crowns, fixed dental prostheses, and implant-retained fixed dental prostheses are examples of CAD/CAM restorations that have been shown to have appropriate marginal adaptability [30]. This knowledge affects how a new plaque forms. When the marginal adaptation is within the range of a clinically tolerable marginal difference, potential caries formation is less likely to happen. Digital technology is replacing the traditional method of taking impressions, which helps to expedite the process [31] and boost patient satisfaction while maintaining an appropriate level of precision (4 to 80 m for scans with a limited region) [32]. A magnified image of the prosthetic substrate is immediately displayed on the computer screen when an intraoral scanner scans the oral cavity, which aids in managing the preparation process and organizing the upcoming restoration. For many operators, this is a major advantage. A recent study done by Bechir et al. aimed to evaluate the corrosion behavior in artificial saliva with different pH values of two commercial cobalt-chromium (Co-Cr) dental alloys manufactured by casting and by milling [33]. They observed that although both cast and milled Co-Cr alloys presented a poorer corrosion resistance in artificial saliva with a more acidic pH value, the milled Co-Cr alloy had better corrosion behavior, making this alloy a better option for the prosthetic treatment of patients. Although not exactly associated, the intervention mentioned in this study (i.e. saliva) was the same used in the investigation by Savencu et al. [17]. The computer-assisted processing technologies showed promising results in this study, representing a good alternative to traditional manufacturing methods for metallic frameworks for dental prostheses. So it can be hypothesized that if the Co-Cr alloys were milled using the CAD/CAM process in the study done by Bechir et al., they might have shown slightly better resistance to corrosion [33]. Literature also shows that technology enables the use of new materials for prosthetic restoration while maintaining process quality control [34]. All of these benefits of CAD/CAM technology can be seen in how happy patients are and how well ceramic and composite restorations work in the long run [35-37].

Some limitations of our review need to be discussed. For example, it is debatable to assume that using CAD-CAM in clinical practice is essential because the majority of the research studies included in our study were in vitro experiments, but, since most of the studies focused on demonstrating the accuracy and precision of CAD/CAM technology in the manufacturing of dental instruments/devices that performed better than their traditional counterparts, the authors believe that in the near future CAD/CAM usage would enhance clinical practice by delivering better quality components and theoretically enhance patient safety as well. Also, the duration of our included studies might be smaller in comparison to what might be considered optimal. Hence, further studies documenting the usage of CAD/CAM technology in dentistry need to be carried out to confirm whether the benefits of CAD/CAM would bring about a significant change in patient convenience and safety in the long run.

## Conclusions

CAD/CAM in different dental specializations has created new opportunities for providing high-quality dental care, at the same time theoretically enhancing patient convenience by enhancing the quality of instruments/devices used. As shown, CAD/CAM technology can be used to create precise and effective dental components that can provide high-quality care. Dentists and patients alike still struggle with the cost of these operations, especially in developing countries. We believe that CAD/CAM will soon be widely employed in all areas of dentistry.
